# Neighborhood income inequality, maternal relative deprivation and neonatal health in Sweden: A cross-sectional study using individually defined multi-scale contexts

**DOI:** 10.1016/j.ssmph.2024.101745

**Published:** 2024-12-29

**Authors:** Per Kåks, Mats Målqvist, Håkan Forsberg, Andreas Alm Fjellborg

**Affiliations:** a Centre for Health and Sustainability, Department of Women's and Children's Health, Uppsala University, Uppsala, Sweden; b Department of Education, Uppsala University, Uppsala, Sweden; c Institute for Housing and Urban Research, Uppsala University, Uppsala, Sweden

**Keywords:** Pregnancy, Birth weight, Prematurity, Socioeconomic status, Social determinants of health

## Abstract

• Maternal relative deprivation is linked to intrauterine growth restriction.
• Neighborhood income inequality is linked to fewer low Apgar scores in high-income mothers.
• Findings support relative deprivation hypothesis over income inequality hypothesis.

## 1 Introduction

The social conditions in which people are born, live and age significantly influence their health throughout all stages of life (Marmot et al., 2008). In public health literature, income as a social determinant of health is commonly conceptualized through three hypotheses: the absolute income hypothesis, the income inequality hypothesis, and the relative deprivation hypothesis (Deaton, 2001; Pickett & Wilkinson, 2015; San Sebastián et al., 2018).

Extensive research has demonstrated how absolute income levels correlate with a range of measures of health and well-being (Marmot et al., 2008). This can be partly explained by the fact that high income enables healthy lifestyles, but also by the psychological security that good finances bring. Additionally, individuals with a higher income are also more likely to utilize preventative services (Devaux, 2015).

The *income inequality hypothesis* posits that the distribution of income within a social context affects health, independent of absolute income levels, with high levels of equality thus being associated with good health and well-being (Pickett & Wilkinson, 2015). Epidemiological studies have indicated that income inequality is linked to a range of measures of poor health, including general mortality, lower life expectancy, risk factors for cardiovascular diseases and mental ill-health (Brodish & Hakes, 2016; Kim et al., 2008; Kawachi & Subramanian, 2018; Patel et al., 2018; Pickett & Wilkinson, 2015; Ribeiro et al., 2017; Zhao et al., 2021; Zheng, 2012).

Conversely, the *relative deprivation hypothesis* suggests that the detrimental effects of income distribution primarily impact those at the bottom of the income hierarchy. Previous studies have associated relative deprivation with outcomes such as poor self-rated health, increased psychiatric symptomatology and higher prevalence of non-communicable disease risk factors (Daly et al., 2015; Mishra & Nicholas Carleton, 2015; Pak & Choung, 2020; Subramanyam et al., 2009; Wetherall et al., 2015).

There are several mechanisms that could explain how income inequality and relative deprivation have adverse effects on health. Large income disparities could lead to a psychosocial burden in the form of chronic stress. This, in turn, could be mediated by lower social capital, reduced social cohesion, and a sense of violations of norms of fairness (Pickett & Wilkinson, 2015; Wilkinson & Pickett, 2006; Wilkinson & Pickett, 2017; Buttrick & Oishi, 2017; Shimonovich et al., 2024). In line with this explanatory model, some research has pointed towards a subjective dimension of the link between inequality and health, where associations between income inequality and poor health are dependent on how income gaps are perceived (Gugushvili et al., 2020; Willis et al., 2022). If reduced social capital and social cohesion mediate the relationship between income inequality and poor health, their absence could potentially impact individuals across all income levels, as these constitute crucial societal resources (Kawachi & Subramanian, 2018; Pickett & Wilkinson, 2015). Certain elements of social cohesion are materially rooted, as societies with lower levels of cohesion often exhibit weaker community organizations and reduced access to support services (Chan et al., 2006). A psychosocial stress response to income disparities could also be mediated by a sense of inferiority through social comparison, which would put individuals at the lower end of the income spectrum at the highest risk (Smith & Huo, 2014; Smith et al., 2012; Daly et al., 2015). Some biomedical research supports the notion that income distribution is linked to a stress response, showing significantly altered levels of stress hormones among relatively deprived individuals, even when controlling for absolute income (Parker et al., 2017; Serwinski et al., 2016). The psychosocial stress associated with income disparities could also lead some individuals to respond with detrimental coping behaviors, such as smoking or alcohol consumption (Smith et al., 2012). Another possible link between income disparities and health is reduced purchasing power among individuals who are deprived in relation to their neighbors. This can render healthy lifestyle choices, such as quality food and use of recreational facilities, less accessible. Income inequality can also lead to under-investments in social goods such as health, education, and community services (Kawachi & Kennedy, 1999).

The association between relative deprivation and health varies depending on how relative deprivation is measured. Measures of relative deprivation include those emphasizing upward social comparisons (e.g. the Yitzhaki index), bidirectional comparisons (e.g. the Deaton index), income ranking methods (e.g. percentile-based measures) and assessments of subjective social status. Although different measures often show overlapping associations with health outcomes, they capture distinct aspects of deprivation that can differentially affect health. Generally, upward-focused measures are more closely linked to poorer mental health outcomes, such as anxiety and depression (Adjaye-Gbewonyo & Kawachi, 2012). In contrast, bidirectional and rank-based measures are often associated with broader physical health indicators, including mortality (Deaton, 2001). Subjective social status, on the other hand, consistently correlates with self-rated health and life satisfaction (Singh-Manoux et al., 2005). Therefore, upward comparisons are more strongly associated with mental health impacts, while bidirectional or rank-based comparisons tend to be linked to overall health outcomes.

The relationship between income inequality, relative deprivation, and health can vary across different geographical scales. Consequently, it is valuable to assess such relationships at multiple scales (Chen & Carol, 2012). Studies based on different levels of aggregation have shown that income inequality within neighborhoods often do not correlate with ill-health in the same way as when inequality is measured at the municipal or regional level (Erdem et al., 2019; Rostila et al., 2012). In contrast to research on state- or nation-level aggregates, higher income inequality has occasionally been associated with better health outcomes when examined at smaller geographical units (D. Kim et al., 2018; Bjornstrom, 2011; Clough-Gorr et al., 2015; Fone et al., 2013; Ghaly & Jivraj, 2022). Possible explanations for this phenomenon include affluent neighborhoods with high inequality demanding better local services, higher-income individuals serving as role models, social comparison not taking place between individuals with extremely large income differences and diverse local contexts indicating social integration and equality at higher levels (Bjornstrom, 2011; Clough-Gorr et al., 2015; Rostila et al., 2012).

Research findings may also differ based on whether health is measured at the individual or aggregate level. In line with this, a study on income inequality and mortality in Sweden found a significant correlation at the individual level but not when outcomes were measured in terms of total mortality at the municipal level (Edvinsson et al., 2013). Such discrepancies highlight the need for precise methods to assess the contextual effects of income distribution and relative income, and problematizes the use of aggregate data. Much of the current evidence on neighborhood-level income inequality and relative deprivation relies on predefined administrative units, suggesting a methodological advantage in using individualized approaches to describe contexts.

Neonatal health offers a valuable lens through which to examine the effects of income inequality and relative deprivation, as early-life health outcomes are particularly sensitive to social and economic disparities. Indicators of neonatal health, such as birth weight, gestational age, and Apgar scores, reflect the influence of maternal socioeconomic conditions and related stressors. Research has linked socioeconomic disadvantage and psychosocial stress, often exacerbated by low relative income, to adverse neonatal outcomes. These associations may be explained by mechanisms such as altered maternal health behaviors, stress-induced hormonal responses, and barriers to prenatal care access (Beijers et al., 2014; Bussières et al., 2015; Clemens & Dibben, 2017). These neonatal outcomes are associated with long-term developmental trajectories that impact health and well-being across the life course (Marmot et al., 2008; Robinson & Martine, 2015). Thus, assessing how neighborhood income inequality and maternal relative deprivation relate to neonatal health contributes to a more nuanced understanding of how socioeconomic inequalities shape health from birth onward.

In recent years, Sweden has experienced widening income gaps, motivating studies on how inequality and relative deprivation impact health (The Lancet Regional Health – Europe, 2023). While previous research in Sweden has primarily focused on adult populations, studies in other countries have shown associations between income inequality or maternal relative deprivation at the state or regional level with higher risks of low birth weight, intrauterine growth restriction and neonatal mortality (Cubbin et al., 2020; Ehntholt et al., 2020; Fujiwara et al., 2013; Lhila & Simon, 2010; Olson et al., 2010; Reagan et al., 2007; Wallace et al., 2016). A report from the Swedish National Board of Health and Welfare has highlighted how absolute income is associated with lower risks of several neonatal health outcomes, such as preterm birth and being small for gestational age (National Board of Health and Welfare, 2016). However, research on the relationship between income inequality, relative deprivation, and neonatal health in Sweden remains limited, including regarding their associations with neighborhood factors.

Understanding the relative dimensions of neighborhood effects is central to grasping how local socioeconomic conditions influence human health across the life course. This study addresses this gap by examining neighborhood-level income inequality and maternal relative deprivation in relation to neonatal health outcomes in Sweden, using individualized contextual analyses to explore at which neighborhood scales these associations might arise.

### 1.1 Aim

This study aimed to investigate whether neighborhood-level income inequality and maternal relative deprivation are linked to neonatal health outcomes in Sweden using individualized contexts and to identify the neighborhood scales at which such links emerge.

## 2 Methods

### 2.1 Study design and sample

This cross-sectional study utilized register data from two national registers collected for the year 2019. The selection of 2019 was made to ensure that the data were unaffected by the COVID-19 pandemic, as prenatal exposure to SARS-COV-2 has been documented to follow sociodemographic patterns and influence various neonatal health outcomes of interest in this study (Yao et al., 2022, 2023).

Socioeconomic, demographic and geographical data were obtained from Statistics Sweden for all persons aged 18 years or older as of January 1, 2019, who resided continuously in the country throughout that year (*n* = 8,038,085). Birth-related data included both neonatal health outcomes and maternal characteristics and were obtained from the Swedish Medical Birth Register. The register includes around 98% of all births in Sweden and the quality of the data is overall very high (Cnattingius et al., 2023). The dataset comprised all live births of singleton children in 2019 to mothers aged 18 years or older (*n* = 108,525).

### 2.2 Individualized neighborhoods

Using the software EquiPop, a k-nearest neighbors method was employed to generate individualized neighborhood contexts of varying scale for each birthing mother (“EquiPop” 2024). EquiPop enables the use of geographically linked data to describe individual contexts for large populations, based on geographical proximity. The software uses population counts for squares in a geographical grid. To create an individualized neighborhood centered on a specified geographical point (i.e. where the individual resides), the people living in the surrounding squares are counted. These squares are successively included in the calculations based on their proximity to the specified point. The inclusion of squares continues until the population count (k) reaches at least the specified k-level, i.e. k = 100, k = 400 etc. In cases where the number of people in a square is larger than required to reach the requested neighborhood size level, all people in that square are included. This means that in some cases the number of people in the neighborhood may be more than specified, and the specified neighborhood sizes are therefore approximate (for applications in other studies, see for example (Türk & Östh, 2019, 2023)).

Income data and household coordinates, approximated based on a grid of 100 × 100 m squares, were collected for all individuals aged 18 and older Sweden. The income data included total disposable income from both labor and capital, minus taxes and negative transfers such as student loan repayments. Income was adjusted based on consumption units in each respective household, which meant that each household's total income was weighted based on the household's composition of adults and children. Disposable income per unit of consumption was used to calculate income percentiles for the total population and to determine percentile standing for each individual.

The EquiPop calculations were performed on four k-levels, generating individualized neighborhoods for each birthing mother consisting of the closest 100, 400, 1600 and 6400 neighbors respectively. In addition, the individuals comprising each neighborhood were also characterized in terms of how many of them belonged to different income percentiles.

### 2.3 Measures of income inequality and relative deprivation

Neighborhood income inequality and maternal relative deprivation measures were derived based on the individualized neighborhoods and the income percentiles of the neighbors. Income inequality was quantified using the Gini index, a concentration index based on the Lorenz curve, which plots the cumulative percentage of total income against the cumulative percentage of the population (Gastwirth, 1972). The Gini index ranges from 0 (indicating perfect income equality) to 1 (indicating perfect income inequality, with one individual holding all the income). A low Gini index can mean a high concentration of a single or a few adjacent income percentiles either in the top, middle or bottom of the distribution.

Relative economic deprivation was assessed using the Deaton index, a variant of the Yitzhaki index (Adjaye-Gbewonyo & Kawachi, 2012; Deaton, 2001). The Yitzhaki index is defined as the cumulative difference between an individual's income and the incomes of those with higher incomes in a reference group, divided by the total number of people in the reference group (Yitzhaki, 1979). The Yitzhaki index is affected by the size of the incomes, and a doubling of all incomes also means doubling the resulting index value. This makes it difficult to use the Yitzhaki index for comparisons between groups such as different neighborhoods. To enable such comparisons, Deaton has proposed a variant of the Yitzhaki index where it is normalized by dividing the index value with the mean income of the reference group (Deaton, 2001). The resulting Deaton index ranges from 0 to 1, with higher values indicating higher relative deprivation. While the Gini index is common to the individuals in a given population, the Deaton index offers an individually unique value that describes an individual's economic position relative to others in the population. The mean Deaton index in a population equals the Gini index of the same population (Adjaye-Gbewonyo & Kawachi, 2012).

### 2.4 Covariates

Individual-level socioeconomic and demographic covariates included maternal age, educational level, disposable income per consumption unit, occupational type (according to the Swedish Standard Classification of Occupations 2012), civil status, region of origin and parity. The distance to the closest labor ward was calculated for each mother based on maternal household coordinates and the locations of labor wards operating in 2019 (National Board of Health and Welfare, 2022). Neighborhood population density, measured in the form of mean distance to neighbors, was calculated at all four k-levels. Mean neighborhood income was also calculated at all four k-levels to control for the influence of increased environmental material standards in affluent neighborhoods.

### 2.5 Neonatal outcomes

Neonatal health was measured using five variables: incidence of preterm birth, low birth weight, very low birth weight, small for gestational age and low Apgar score at 5 min post-birth. The Apgar score is a composite measure of newborn vitality ranging from 0 to 10, with scores <7 indicating a need for medical attention. Preterm birth was defined as birth before gestational week 37. Low birthweight and very low birth weight was defined as <2500 g and <1500 g respectively. Small for gestational age was defined as birth weight below the 10th percentile for gestational age. All health outcomes were dichotomized according to these cutoff points.

### 2.6 Statistical analyses

Descriptive statistics were used to characterize the population. Multiple logistic regression was employed to investigate the associations between neighborhood income inequality, maternal relative deprivation, and neonatal health outcomes. Analyses were conducted for both the Gini and Deaton indices at all four neighborhood scales. For each neighborhood scale, the mean neighborhood income was included as a covariate along with individual-level characteristics.

Average Marginal Effects (AMEs) and 95% confidence intervals (95% CIs) were calculated for increments of 0.1 of both indices. Each combination of neighborhood scale, index, and neonatal health outcome was analyzed, resulting in 80 regression models. Interaction effects between indices and individual income were examined by repeating AME analyses with mothers divided into income deciles. Missing data were excluded from the analysis. All analyses were performed using RStudio (version 2021.09.0).

## 3 Results

### 3.1 Study population

The study included 108,525 live births of singleton children. Maternal socioeconomic and demographic characteristics, along with rates of neonatal health outcomes by these characteristics, are shown in Table 1. Approximately 30% of mothers were born in another country, the majority of which were born in a country not belonging to the Organisation for Economic Co-operation and Development (OECD). The majority of mothers were employed, while 21.2% were unemployed or had an unregistered occupation. Approximately 42% were primiparous. The proportion of children born with low birthweight were 3.1% and 0.6% fulfilled criteria for very low birth weight. Preterm birth constituted 4.8% of all births and 2.4% of newborns were classified as small for gestational age. Low Apgar score had been registered in 1.6% of all cases. Overall, the rates of adverse neonatal outcomes were higher in groups with lower educational attainment. The patterns in this data were also consistent with what would be expected from other socioeconomic and demographic variables.

**Table 1:** Maternal socioeconomic and demographic characteristics and rates of neonatal health outcomes.

Characteristic	Total count	Neonatal health outcomes				
		Small for gestational age	Low birth weight	Very low birth weight	Preterm birth	Low Apgar score
Total	108,525 (100.0%)	2596 (2.4%)	3314 (3.1%)	590 (0.5%)	5115 (4.7%)	1687 (1.6%)
*Region of origin*						
Sweden	76,160 (70.2%)	1546 (2.0%)	2191 (2.9%)	380 (0.5%)	3644 (4.8%)	1152 (1.5%)
Nordic countries, not including Sweden	1196 (1.1%)	18 (1.5%)	30 (2.5%)	6 (0.5%)	48 (4.0%)	20 (1.7%)
OECD, not including Nordic countries	5279 (4.9%)	132 (2.5%)	165 (3.1%)	24 (0.5%)	241 (4.6%)	61 (1.2%)
Outside OECD	25,890 (23.9%)	900 (3.5%)	928 (3.6%)	180 (0.7%)	1182 (4.6%)	454 (1.8%)
*Education*						
Basic education <9 years	3966 (3.7%)	143 (3.6%)	147 (3.7%)	30 (0.8%)	194 (4.9%)	88 (2.2%)
Basic education ≥9 years	6860 (6.3%)	225 (3.3%)	293 (4.3%)	47 (0.7%)	405 (5.9%)	144 (2.1%)
Upper secondary education ≤2 years	7804 (7.2%)	240 (3.1%)	296 (3.8%)	55 (0.7%)	413 (5.3%)	158 (2.0%)
Upper secondary education 3 years	28,860 (26.6%)	635 (2.2%)	917 (3.2%)	174 (0.6%)	1512 (5.2%)	446 (1.5%)
Tertiary education <3 years	15,477 (14.3%)	370 (2.4%)	459 (3.0%)	80 (0.5%)	692 (4.5%)	208 (1.3%)
Tertiary education ≥3 years	42,367 (39.0%)	880 (2.1%)	1107 (2.6%)	193 (0.5%)	1776 (4.2%)	599 (1.4%)
Doctoral education	930 (0.9%)	23 (2.5%)	18 (1.9%)	1 (0.1%)	21 (2.3%)	10 (1.1%)
Unregistered	2261 (2.1%)	80 (3.5%)	77 (3.4%)	10 (0.4%)	102 (4.5%)	34 (1.5%)
*Occupational type*						
Unemployed or unregistered occupation	22,957 (21.2%)	751 (3.3%)	832 (3.6%)	125 (0.5%)	1154 (5.0%)	383 (1.7%)
Military	47 (0.0%)	0 (0.0%)	1 (2.1%)	0 (0.0%)	2 (4.3%)	1 (2.1%)
Manager	2709 (2.5%)	57 (2.1%)	75 (2.8%)	15 (0.6%)	112 (4.1%)	43 (1.6%)
Occupations requiring advanced level of higher education	29,878 (27.5%)	605 (2.0%)	783 (2.6%)	135 (0.5%)	1274 (4.3%)	424 (1.4%)
Occupations requiring higher education qualifications or equivalent	10,291 (9.5%)	184 (1.8%)	247 (2.4%)	44 (0.4%)	435 (4.2%)	120 (1.2%)
Administration and customer service clerk	7284 (6.7%)	154 (2.1%)	191 (2.6%)	39 (0.5%)	340 (4.7%)	100 (1.4%)
Service, care and shop sales worker	27,624 (25.5%)	643 (2.3%)	906 (3.3%)	175 (0.6%)	1396 (5.1%)	489 (1.8%)
Agricultural, horticultural, forestry and fishery worker	526 (0.5%)	9 (1.7%)	14 (2.7%)	4 (0.8%)	27 (5.1%)	12 (2.3%)
Building and manufacturing worker	1139 (1.0%)	27 (2.4%)	41 (3.6%)	9 (0.8%)	62 (5.4%)	14 (1.2%)
Mechanical manufacturing and transport worker	1785 (1.6%)	32 (1.8%)	60 (3.4%)	16 (0.9%)	94 (5.3%)	24 (1.3%)
Elementary occupations	4285 (3.9%)	134 (3.1%)	164 (3.8%)	28 (0.7%)	219 (5.1%)	77 (1.8%)
*Civil status*						
Unmarried	53,322 (49.1%)	1230 (2.3%)	1645 (3.1%)	306 (0.6%)	2590 (4.9%)	853 (1.6%)
Married	50,629 (46.7%)	1224 (2.4%)	1479 (2.9%)	249 (0.5%)	2243 (4.4%)	745 (1.5%)
Divorced	4426 (4.1%)	138 (3.1%)	185 (4.2%)	35 (0.8%)	274 (6.2%)	85 (1.9%)
Widowed	148 (0.1%)	4 (2.7%)	5 (3.4%)	0 (0.0%)	8 (5.4%)	4 (2.7%)
*Age*						
<25 years	10,704 (9.9%)	314 (2.9%)	359 (3.4%)	54 (0.5%)	534 (5.0%)	181 (1.7%)
25–29 years	34,471 (31.8%)	750 (2.2%)	969 (2.8%)	169 (0.5%)	1577 (4.6%)	547 (1.6%)
30–34 years	38,875 (35.8%)	873 (2.2%)	1108 (2.9%)	198 (0.5%)	1743 (4.5%)	544 (1.4%)
35–39 years	19,632 (18.1%)	499 (2.5%)	646 (3.3%)	125 (0.6%)	940 (4.8%)	307 (1.6%)
≥40 years	4843 (4.5%)	160 (3.3%)	232 (4.8%)	44 (0.9%)	321 (6.6%)	108 (2.2%)
*Parity, including current birth*						
1	45,964 (42.4%)	1620 (3.5%)	1909 (4.2%)	352 (0.8%)	2636 (5.7%)	965 (2.1%)
2	40,348 (37.2%)	595 (1.5%)	814 (2.0%)	137 (0.3%)	1445 (3.6%)	424 (1.1%)
3	14,928 (13.8%)	225 (1.5%)	349 (2.3%)	63 (0.4%)	593 (4.0%)	183 (1.2%)
4	4475 (4.1%)	103 (2.3%)	154 (3.4%)	21 (0.5%)	259 (5.8%)	67 (1.5%)
≥5	2810 (2.6%)	53 (1.9%)	88 (3.1%)	17 (0.6%)	182 (6.5%)	48 (1.7%)
*Average distance to closest 100 neighbors*						
0–99 m	43,257 (39.9%)	1222 (2.8%)	1449 (3.3%)	267 (0.6%)	2035 (4.7%)	696 (1.6%)
100–499 m	51,138 (47.1%)	1114 (2.2%)	1484 (2.9%)	248 (0.5%)	2375 (4.6%)	746 (1.5%)
≥500 m	14,129 (13.0%)	260 (1.8%)	381 (2.7%)	75 (0.5%)	705 (5.0%)	245 (1.7%)
*Individual disposable income*						
0-99,999 SEK/year	17,358 (16.0%)	595 (3.4%)	659 (3.8%)	98 (0.6%)	900 (5.2%)	297 (1.7%)
100,000–199,999 SEK/year	55,458 (51.1%)	1172 (2.1%)	1563 (2.8%)	271 (0.5%)	2474 (4.5%)	846 (1.5%)
200,000–299,999 SEK/year	28,284 (26.1%)	648 (2.3%)	840 (3.0%)	172 (0.6%)	1363 (4.8%)	439 (1.6%)
300,000–399,999 SEK/year	5191 (4.8%)	125 (2.4%)	168 (3.2%)	31 (0.6%)	254 (4.9%)	80 (1.5%)
400,000–499,999 SEK/year	1242 (1.1%)	31 (2.5%)	59 (4.8%)	12 (1.0%)	78 (6.3%)	17 (1.4%)
≥500,000 SEK/year	992 (0.9%)	25 (2.5%)	25 (2.5%)	6 (0.6%)	46 (4.6%)	8 (0.8%)
*Mean disposable income among closest 100 neighbors*						
0-99,999 SEK/year	1884 (1.7%)	73 (3.9%)	76 (4.0%)	9 (0.5%)	91 (4.8%)	42 (2.2%)
100,000–199,999 SEK/year	79,194 (73.0%)	1982 (2.5%)	2531 (3.1%)	446 (0.6%)	3869 (4.9%)	1301 (1.6%)
200,000–299,999 SEK/year	25,922 (23.9%),	513 (2.0%)	672 (2.6%)	131 (0.5%)	1084 (4.2%)	333 (1.3%)
300,000–399,999 SEK/year	1398 (1.3%)	25 (1.8%)	32 (2.3)%	4 (0.3%)	66 (4.7%)	11 (0.8%)
400,000–499,999 SEK/year	123 (0.1%)	3 (2.4%)	3 (2.4%)	0 (0.0%)	5 (4.1%)	0 (0.0%)
≥500,000 SEK/year	4 (0.0%)	0 (0.0%)	0 (0.0%)	0 (0.0%)	0 (0.0%)	0 (0.0%)
*Distance to closest labor ward*						
0–19 km	74,228 (68.4%)	1844 (2.5%)	2277 (3.1%)	405 (0.5%)	3401 (4.6%)	1061 (1.4%)
20–39 km	22,057 (20.3%)	481 (2.2%)	658 (3.0%)	118 (0.5%)	1072 (4.9%)	376 (1.7%)
40–59 km	8269 (7.6%)	186 (2.2%)	260 (3.1%)	50 (0.6%)	433 (5.2%)	172 (2.1%)
60–79 km	2261 (2.1%)	47 (2.1%)	73 (3.2%)	10 (0.4%)	118 (5.2%)	48 (2.1%)
80–99 km	1137 (1.0%)	22 (1.9%)	34 (3.0%)	6 (0.5%)	61 (5.4%)	20 (1.8%)
≥100 km	573 (0.5%)	16 (2.8%)	12 (2.1%)	1 (0.2%)	30 (5.2%)	10 (1.7%)

### 3.2 Associations with neonatal health outcomes

**Fig. 1:**
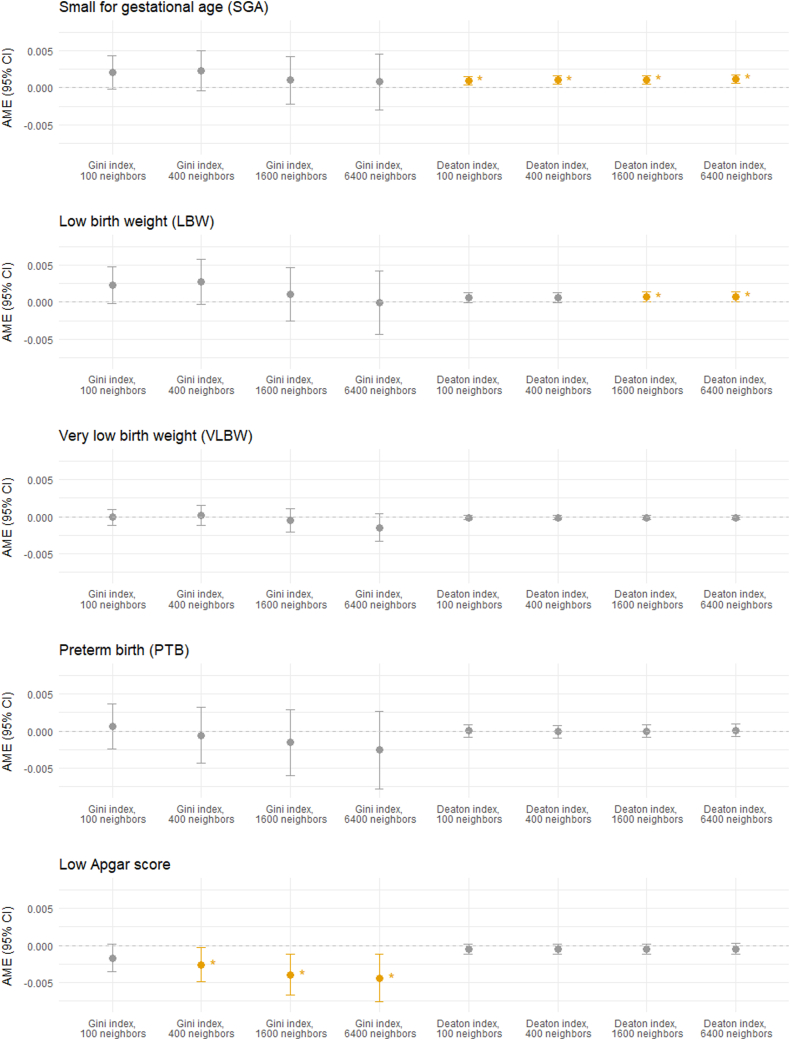
Visualization of associations between Gini and Deaton indices and neonatal health outcomes. All associations are shown for 0.1 increases of indices. Statistically significant (95% CI) Average Marginal Effects (AME) and 95% Confidence Intervals (p < 0.05) are colored and marked with an asterisk (∗).

The associations between Gini and Deaton indices and neonatal health outcomes obtained from the regression models are summarized in Table 2 and visualized in Fig. 1. Detailed results for all variables in each regression model can be found in Additional file 1.

**Table 2:** Associations between Gini and Deaton indices and neonatal health outcomes. Increased Gini and Deaton indices indicate higher neighborhood income inequality and higher level of maternal financial disadvantage compared to their neighbors, respectively. All associations are shown for 0.1 increases of indices, both having a maximum range of 0–1. Statistically significant (p < 0.05) Average Marginal Effects (AME) and 95% Confidence Intervals (95% CI) are highlighted in bold.

Index	Neighborhood scale	Neonatal health outcome									
		Small for gestational age (<10th percentile)		Low birth weight (<2500 g)		Very low birth weight (<1500 g)		Preterm birth (gestational week <37)		Low Apgar score (<7)	
		AME	95% CI	AME	95% CI	AME	95% CI	AME	95% CI	AME	95% CI
Gini index (neighborhood income inequality, 0.1 increment)	100 neighbors	0.00205	−0.00016, 0,0.00427	0.00232	−0.00016, 0.00480	−0.00007	−0.00115, 0.00100	0.00058	−0.00246, 0.00361	−0.00167	−0.00350, 0.00016
	400 neighbors	0.00232	−0.00039, 0.00504	0.00276	−0.00029, 0.00580	0.00015	−0.00116, 0.00147	−0.00057	−0.00433, 0.00318	**−0.00257**	**−0.00486, -0.00028**
	1600 neighbors	0.00103	−0.00217, 0.00424	0.00104	−0.00256, 0.00465	−0.00046	−0.00202, 0.00111	−0.00153	−0.00598, 0.00292	**−0.00392**	**−0.00667, -0.00117**
	6400 neighbors	0.00081	−0.00295, 0.00457	−0.00006	−0.00431, 0.00419	−0.00147	−0.00336, 0.00041	−0.00254	−0.00778, 0.00270	**−0.00436**	**−0.00760, -0.00112**
Deaton index (maternal relative deprivation, 0.1 increment)	100 neighbors	**0.00098**	**0.00042, 0.00153**	0.00061	−0.00005, 0.00126	−0.00015	−0.00044, 0.00014	0.00003	−0.00081, 0.00086	−0.00050	−0.00116, 0.00016
	400 neighbors	**0.00103**	**0.00047, 0.00158**	0.00063	−0.00004, 0.00129	−0.00013	−0.00042, 0.00016	−0.00006	−0.00090, 0.00079	−0.00048	−0.00114, 0.00018
	1600 neighbors	**0.00110**	**0.00054, 0.00166**	**0.00068**	**0.00001, 0.00135**	−0.00012	−0.00041, 0.00018	−0.00001	−0.00087, 0.00088	−0.00045	−0.00112, 0.00021
	6400 neighbors	**0.00120**	**0.00064, 0.00176**	**0.00074**	**0.00007, 0.00141**	−0.00013	−0.00043, 0.00016	0.00011	−0.00074, 0.00096	−0.00043	−0.00111, 0.00025

Neighborhood income inequality, as measured by the Gini index, showed significant negative associations with the incidence of low Apgar score when calculated based on the 400 (AME: 0.00257, 95% CI: −0.00486, −0.00028), 1600 (AME: 0.00392, 95% CI: −0.00667, −0.00117), and 6400 closest neighbors (AME: 0.00436, 95% CI: −0.00760, −0.00112). No significant associations were observed between income inequality and low birth weight, very low birth weight, preterm birth, or being small for gestational age.

Maternal relative deprivation, as measured by the Deaton index, was significantly associated with both low birth weight across the two larger neighborhood sizes. A 0.1 increase in the Deaton index had an AME of 0.00068 (95% CI: 0.00001, 0.00135) when calculated based on neighborhoods consisting of the 1600 closest neighbors and an AME of 0.00074 (95% CI: 0.00007, 0.00141) when considering the 6400 closest neighbors.

Being small for gestational age was more prevalent among children of relatively deprived mothers, with 0.1 increases in the Deaton index having AMEs of 0.00098 (95% CI: 0.00042, 0.00153) for neighborhoods of 100 closest neighbors, 0.00103 (95% CI: 0.00047, 0.00158) for 400 neighbors, 0.00110 (95% CI: 0.00054, 0.00166) for 1600 neighbors, and 0.00120 (95% CI: 0.00064, 0.00176) for 6400 neighbors. No significant associations were found between the Deaton index and very low birth weight, preterm birth, or low Apgar scores at any neighborhood scale.

In summary, among every 100 newborns, 0.68–0.74 more were born with low birth weight, and 0.98–1.20 more were born small for gestational age to the most relatively deprived mothers (Deaton index = 1) compared to the least relatively deprived mothers (Deaton index = 0).

### 3.3 Interaction effects between indices and maternal income

**Fig. 2:**
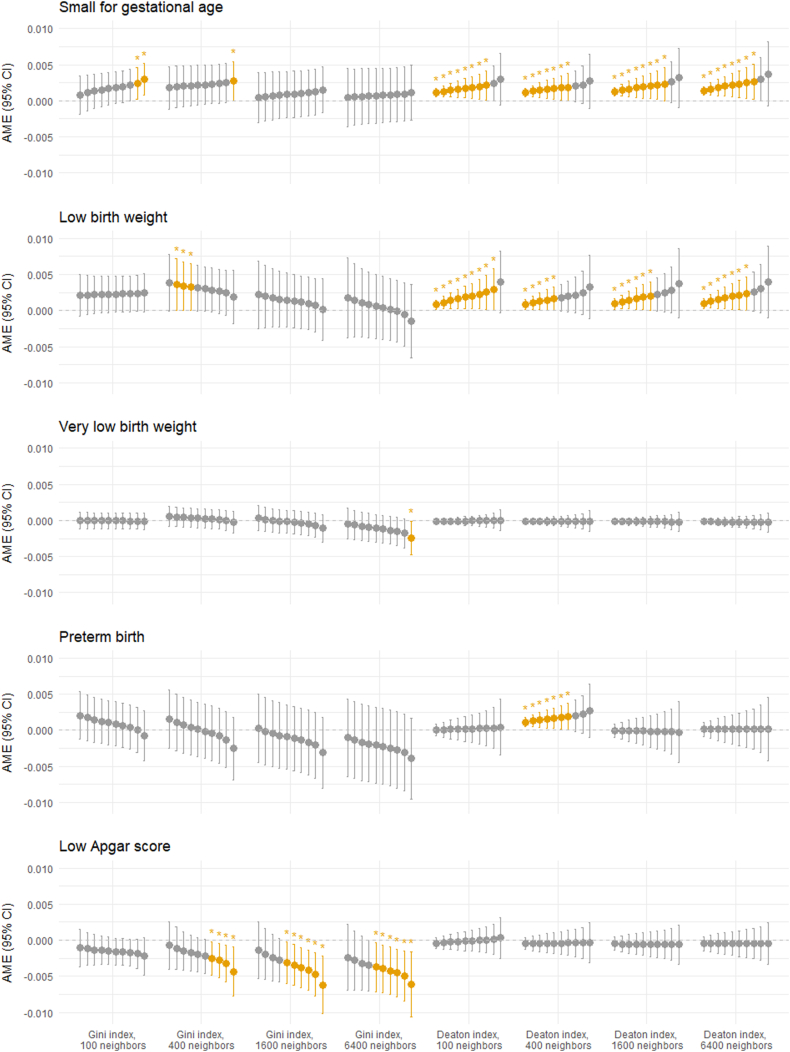
Visualizations of associations between Gini and Deaton indices and neonatal health outcomes across maternal income deciles. All associations are shown for 0.1 increases of indices. Each point and associated whiskers represent an income decile, with lower deciles to the left and higher to the right. Statistically significant (p < 0.05) Average Marginal Effects (AME) and 95% Confidence Intervals (95% CI) are colored and marked with an asterisk (∗).

Regression models incorporating interaction effects between indices and individual maternal income are visualized in Fig. 2, with detailed results for all indices available in Additional File 2. Results for all independent variables are available in Additional File 3. These models revealed that the negative association between the Gini index and the incidence of low Apgar scores was significant for children to mothers in the 4 highest income deciles for neighborhoods consisting of 400 individuals and the 6 highest income deciles for neighborhoods consisting of 1600 and 6400 individuals. Conversely, the associations for children to mothers in lower income deciles were not statistically significant.

A high Deaton index was more strongly associated with low birth weight and small for gestational age among newborns of mothers with high absolute income than among those with low, in terms of point estimates measured at all neighborhood scales. Among the highest income deciles, these point estimates were accompanied by large confidence intervals that made the associations non-significant.

## 4 Discussion

This study investigated how neighborhood-level income inequality and maternal relative deprivation links to neonatal health outcomes in Sweden, using individualized contexts across multiple scales. The findings indicate that maternal relative deprivation is significantly associated with giving birth to growth-restricted children, as measured by both low birth weight and being small for gestational age. The findings also highlight that high-income mothers living in neighborhoods with high income inequality give birth to children with significantly lower rates of low Apgar scores than those living in neighborhoods with higher levels of equality. Overall, this study provides some support for the relative deprivation hypothesis while contradicting the income inequality hypothesis. This indicates that living in proximity to individuals with higher income than oneself is associated with worse health outcomes in terms of intrauterine growth among newborns, and that this effect is stronger the higher the income of the birthing mother.

These findings are consistent with the few previous studies on the topic. In the US, a study by Lhila and Simon demonstrated that mothers who were relatively deprived in relation to other mothers in the same metropolitan area gave birth to children with slightly lower birth weights (Lhila & Simon, 2010). A study by Reagan et al. evaluating different measures of relative poverty, also based on US data linked to metropolitan areas, found a higher likelihood of children being growth-restricted among families that were relatively deprived, regardless of measure used (Reagan et al., 2007).

It is well established that birthweight is partially determined both by maternal health and lifestyles and prenatal environmental exposure, often jointly referred to as the pregnancy exposome (Robinson & Martine, 2015). Previous research has linked maternal prenatal stress to higher risks of giving birth to children of low weight, although results have varied depending on stressor and context (Bussières et al., 2015). Studies on neighborhood-level stress-inducing factors such as high crime rates and environmental noise levels have also shown similar associations with low birth weight and being small for gestational age (Clemens & Dibben, 2017; Nieuwenhuijsen et al., 2017). Several biological mechanisms could potentially mediate such links between stress and growth restriction: a hormonal response involving secretion of cortisol could have direct effects on fetal growth, maternal immune functioning and intestinal microbiota can be affected as a consequence, and stress can alter maternal behaviors related to sleep, diet and exercise (Beijers et al., 2014). Such mechanisms are possible explanations for associations between relative deprivation and poor health at birth, but are difficult to explore in register-based studies.

Other maternal risk factors for growth restriction among newborns include lifestyle and health factors such as tobacco, alcohol and drug use (Campbell et al., 2012). Such factors have been demonstrated as mediators in other studies on relative deprivation and health at birth. The study by Lhila and Simon saw higher rates of smoking among relatively deprived mothers, at levels that could explain the higher rates of low birth weight (Lhila & Simon, 2010). Research focusing on other outcomes have demonstrated similar findings. In a study on relative deprivation and self-rated health in Taiwan by Kuo and Chiang, relative deprivation was associated with both smoking and depressive symptoms (Kuo & Chiang, 2013). A study on relative deprivation and chronic diseases in China by Wu et al. (2020) similarly saw increased rates of smoking among relatively deprived individuals, as well as increased rates of alcohol consumption.

In addition to stress and detrimental lifestyle factors, maternal health factors such as hypertension, low body mass index, poor nutritional status and low weight gain during pregnancy also increase the risk of newborns not reaching their expected weight at birth (Baker et al., 2018; Campbell et al., 2012). It is theoretically possible that relative deprivation reflects locally higher prices of both goods and services. However, it is less plausible that such effects emerge on the geographical levels explored in this study as prices for consumer goods and other services are not determined by the purchasing power in such small geographies.

Contrasting with previous research exploring the income inequality hypothesis in relation to neonatal health (Cubbin et al., 2020; Fujiwara et al., 2013; Olson et al., 2010; Wallace et al., 2016), this study showed no significant associations between income inequality and any of the variables for birth weight or preterm birth. As none of these studies included Apgar score as an outcome measure, no comparison can be made with the lower prevalence of low Apgar in neighborhoods with high income inequality in this study. This finding has no apparent explanation. However, high levels of neighborhood income inequality could potentially be theorized to indicate the presence of a few individuals with very high incomes who are in a position to demand better local services such as recreational facilities. If that is the case, those benefiting from such services might disproportionately be high-income mothers, which is in line with our findings. The difference between income groups in the association between income inequality and Apgar scores may also be in line with the notion that the perception of inequalities matters, as those at the top and bottom of the income hierarchy may perceive income gaps differently.

### 4.1 Study strengths and limitations

A strength of this study was the use of the EquiPop software, which represents a methodological contribution to the study of neighborhood effects by enabling the creation of individualized contexts. This approach provides a more precise analysis of links between neighborhood characteristics and health outcomes than what can be accomplished when data such as income distribution is based on predefined administrative units. Additionally, the study benefited from the high quality of data obtained from Statistics Sweden and the Swedish Medical Birth Register.

The study also has limitations. The cross-sectional design limits the ability to draw causal inferences from the observed associations. While the register data is robust, it may not capture the range of relevant variables that could influence neonatal health outcomes, such as psychosocial factors, maternal stress or nutrition. In line with this, this study did not evaluate the different potential mediating pathways. The neighborhoods were based on all adults in Sweden, which does not take into account that social comparisons may be made to different degrees between individuals belonging to different social and age groups. Furthermore, the Deaton index is affected by the incomes both lower and higher than the one of the individual in question, as it includes a division by mean income of the whole reference group. This contrasts with the original notion of relative deprivation first developed by Runciman, which only emphasizes upward social comparison (Runciman, 1966).

## 5 Conclusions

This study highlights that maternal experiences of relative deprivation – essentially being economically worse off than others in the same neighborhood – are associated with adverse neonatal outcomes in the form of having low birth weight and being small for gestational age. Conversely, higher neighborhood income inequality is linked to lower rates of low Apgar scores, but this benefit is primarily seen among high-income mothers. These findings support the relative deprivation hypothesis at the neighborhood level, suggesting that individuals lower in the socioeconomic hierarchy experience worse outcomes when living near those much higher in the same hierarchy. In contrast, they also challenge the income inequality hypothesis, indicating that simply having a high degree of income inequality in a neighborhood is not universally linked to worse health outcomes. In other words, neighborhoods with high income disparities may be detrimental exclusively to those on the lower end of the local income hierarchy. Overall, these findings highlight that social inequities in neonatal health extend beyond what would be predicted by the absolute socioeconomic status of families in Sweden.

## CRediT authorship contribution statement

**Per Kåks:** Writing – original draft, Visualization, Project administration, Methodology, Investigation, Formal analysis, Data curation, Conceptualization. **Mats Målqvist:** Writing – review & editing, Validation, Supervision, Resources, Methodology, Funding acquisition, Conceptualization. **Håkan Forsberg:** Writing – review & editing, Validation, Supervision, Software, Methodology, Investigation, Formal analysis, Data curation, Conceptualization. **Andreas Alm Fjellborg:** Writing – review & editing, Validation, Supervision, Software, Methodology, Investigation, Formal analysis, Data curation, Conceptualization.

## Ethical approval

The Swedish Ethical Review Authority approved of the study (2022-03771-01).

## Data availability

The register data used for this study are not publicly available due to confidentiality regulations governing the use and distribution of research data from Swedish national registers. However, the detailed results from all regression models are available in Additional File 1, Additional File 2 and Additional File 3. The statistical code is available on request from PK.

## Funding

This study was fully funded by the Department of Women's and 10.13039/100012422Children's Health, 10.13039/501100007051Uppsala University.

## Declaration of competing interest

The authors declare no competing interests.
